# Oral Hygiene is Associated with Orthodontic Pain in Patients with Treated and Stabilised Periodontitis

**DOI:** 10.3290/j.ohpd.b2183027

**Published:** 2021-10-22

**Authors:** Fung Hou Kumoi Mineaki Howard Sum, Chong Ren, Min Gu, Lijian Jin, Colman McGrath, Yanqi Yang

**Affiliations:** a AdvDipOrth Candidate, Faculty of Dentistry, The University of Hong Kong, Hong Kong, SAR, China. Performed clinical examinations, wrote the manuscript, proofread the manuscript, performed statistical evaluation.; b Senior Clinical Practitioner in Orthodontics, Faculty of Dentistry, The University of Hong Kong, Hong Kong, SAR, China. Designed and performed experiments (pain recording).; c Clinical Assistant Professor in Orthodontics, Faculty of Dentistry, The University of Hong Kong, Hong Kong, SAR, China. Performed the experiments, proofread the manuscript.; d Clinical Professor in Periodontology, Faculty of Dentistry, The University of Hong Kong, Hong Kong, SAR, China. Experimental design, periodontal recording and proofread the manuscript.; e Clinical Professor in Dental Public Health, Faculty of Dentistry, The University of Hong Kong, Hong Kong, SAR, China. Proofread the manuscript, consulted on statistical evaluation, contributed substantially to discussion.; f Clinical Associate Professor in Orthodontics, Faculty of Dentistry, The University of Hong Kong, Hong Kong, SAR, China. Designed and performed experiments, proofread the manuscript, contributed substantially to discussion.

**Keywords:** oral hygiene, orthodontic treatment, pain, treated and stabilised periodontitis

## Abstract

**Purpose:**

This prospective cohort study aimed to 1) determine whether oral hygiene (OH) is a factor affecting orthodontic pain and 2) reveal whether orthodontic pain affects OH practice during orthodontic treatment.

**Materials and Methods:**

35 adults aged 22–59 years with treated and stabilised periodontitis were recruited. The pre-bonding (baseline) and 1-month post-bonding OH as well as periodontal status were recorded. The experience, duration and maximum intensity of orthodontic pain within the first week after bonding were documented. In addition, the concentrations of cytokines in the gingival crevicular fluid (GCF) were recorded at baseline, 1 day and 1 week after bonding.

**Results:**

Patients who experienced orthodontic pain in the first week of orthodontic treatment had a higher baseline gingival index (GI) than patients who never experienced orthodontic pain (p < 0.05), and patients who experienced a longer duration and higher intensity of orthodontic pain in the first week of orthodontic treatment also had a higher baseline GI (p < 0.05). Patients who experienced orthodontic pain in the first week of orthodontic treatment had statistically significantly higher concentrations of interleukin 1β (IL-1β) in GCF at 1 day post bonding than those who never experienced pain, while baseline GI was positively associated with cytokine concentrations in GCF at 1 week post bonding (p < 0.05). In addition, neither the experience of orthodontic pain nor its duration and intensity were associated with the level of post-bonding OH (p > 0.05).

**Conclusions:**

The finding that increased gingival inflammation accounted for the longer duration and higher intensity of orthodontic pain in treated and stabilised periodontal patient shows that oral hygiene instructions and supportive periodontal care are of great importance prior to and during adjunctive orthodontic treatment in periodontally compromised individuals.

Increasing numbers of adults are seeking orthodontic treatment to straighten teeth and improve bite for aesthetic and functional reasons. Among adults seeking orthodontic treatment, 12% have periodontal disease.^15^ These patients frequently present with drifting incisors, tilted posterior teeth, traumatic overbite and missing teeth caused by periodontal problems.^15,18^ It is believed that orthodontic treatment can improve periodontal and dental health by aligning teeth and balancing dental occlusion. Aligning the teeth allows better access to teeth for cleaning, and thus facilitates and improves oral hygiene maintenance, while balancing dental occlusion can reduce occlusal trauma, making it an important part of periodontal therapy.^2^

Apart from improving dental function, another motivation is the desire to improve the aesthetic appearance of teeth.^15,39^ Pathological tooth migration is an evident sign of periodontitis and the drifting of teeth can affect dentofacial aesthetics. This drifting problem is more common in the anterior dentition due to a lack of stable occlusion and sagittal contacts with the opposing teeth.^12,42^ Aesthetically acceptable results can be achieved by orthodontic tooth movements such as intrusion, rotation and uprighting, to move the teeth into their desired position.^44^

Although orthodontic treatment can improve patients’ occlusion, providing a more attractive smile and greater self-confidence,^19,23,34^ the majority of orthodontic patients (90%) report painful experiences and 30% consider discontinuing treatment prematurely because of pain.^24,32^ Orthodontic pain can be perceived as discomfort, dull pain or hypersensitivity in the affected teeth.^1,21^ The mechanism of orthodontic pain has been investigated in some studies.^28^ Mechanical force applied during orthodontic tooth movement stimulates an increase in the concentrations of cytokines such as prostaglandin E2 (PGE2) and interleukin 1β (IL-1β) in the gingival crevicular fluid (GCF) of the teeth undergoing orthodontic movement.^41,43^ These cytokines released during orthodontic tooth movement may also elicit a hyperalgesic response by binding to sensory nerve endings, generating a painful sensation.^23,28^ Poor oral hygiene (OH) can cause inflammation of the periodontal tissues, while inflammation also leads to increased concentrations of cytokines such as PGE2 and IL-1β. Therefore, it is hypothesised that unsatisfactory baseline oral hygiene may be associated with the experience and level of orthodontic pain. However, pertinent studies in this area are lacking in the literature.

Other than causing orthodontic pain, orthodontic appliances are reported to be plaque retentive.^40^ Patients undergoing fixed orthodontic treatment have more difficulty in maintaining proper OH because the orthodontic wire and brackets impede conventional brushing and flossing. This leaves orthodontic patients at a higher risk of gingivitis.^3^ Without proper maintenance of OH, periodontal destruction will occur or be aggravated during tooth movement.^26^ Maintaining adequate OH is extremely important for patients with periodontitis, as compromised OH will worsen periodontal problems. Unfortunately, discomfort and pain will discourage a person from touching and intervening with the affected area. Sergl et al^35^ found that orthodontic pain was associated with poor overall compliance, where poorer OH was one of the factors included when assessing the overall compliance. However, the study did not establish whether orthodontic pain was directly associated with OH practice. Another study showed that chronic orofacial pain could lead to poor dental hygiene,^11^ but did not investigate orthodontic pain. To date, no study has directly explored the effect of orthodontic pain on OH maintenance. More research is therefore required on whether orthodontic pain affects post-bonding OH maintenance.

In light of the above research gaps, it would be valuable to know whether patients’ OH at the beginning of orthodontic treatment affects their level of orthodontic pain during treatment, and whether their experience of pain during treatment will affect their post-bonding OH maintenance, in the context of treated and stabilised periodontitis.

The aim of the study was to investigate the association between OH and orthodontic pain in patients with treated and stabilised periodontitis, with the following objectives. The first objective was to investigate the association between the baseline level of OH and that of orthodontic pain during orthodontic treatment, to determine whether OH is a factor affecting orthodontic pain. The second objective was to investigate the association between the experience of orthodontic pain and the post-bonding OH status, to determine whether orthodontic pain affects OH practice during orthodontic treatment.

## Materials and Methods

Ethical approval was obtained from the Institutional Review Board of the University of Hong Kong/Hospital Authority Hong Kong West Cluster (HKU/HA HKW IRB, Ref. No. UW 12-049).

### Sample

According to a previous study investigating how orthodontic tooth movements affect periodontal tissues^7^ and a study on pain and orthodontic treatment,^24^ the standard deviation (SD) of the gingival index (GI) may be 0.237 and the pain allocation ratio may be 2:1.^24^ Therefore, a total sample size of 30 would have 80% power to detect a clinically statistically significant difference of GI (defined as 0.25) at the 0.05 significance level, using G*Power version 3.1.9.2 (Franz Faul, University of Kiel, Kiel, Germany).

This prospective cohort study therefore recruited 35 Chinese adult patients (29 women, 6 men, aged 22–59 years) with treated and stabilised periodontitis. The patients were recruited from Prince Philip Dental Hospital (PPDH), the only dental hospital in Hong Kong, and informed consent was signed before the treatment. The inclusion criteria required that the patients be systemically healthy adults who had not taken any antibiotic and/or anti-inflammatory drugs in the previous month. They were diagnosed with periodontitis with a pocket depth >5 mm, clinical attachment loss >3 mm and radiographic bone loss before non-surgical periodontal treatment. Before orthodontic treatment, all periodontal pocket depths were required to be less than 4 mm and the periodontal status had to have been stable for at least 3 months after non-surgical periodontal treatment.

The exclusion criteria were patients who smoked, were pregnant, had previous orthodontic treatment, or had previous periodontal surgery.

### Orthodontic Treatment

All of the orthodontic treatments were performed by the same orthodontist at the Faculty of Dentistry, the University of Hong Kong. A preadjusted appliance with 0.022 x 0.028-inch brackets/buccal tubes (3M Unitek MBT Versatile + Appliance System; Landsberg, Germany) was used. All patients had the same standardised archwire of 0.014-inch thermal NiTi (G&H Orthodontics, M5 Thermal Copper Nickel Titanium; Franklin, IN, USA) as the initial archwire for the first month of orthodontic treatment.

### Outcome Assessments

Orthodontic treatment, periodontal examination, pain assessment and GCF sample collection were conducted by different blinded operators. Throughout orthodontic treatment, the blinded operators were not aware of the treatment conducted or the results collected by the other operators.

#### Periodontal examination

All of the periodontal charting was carried out by the same dentist and calibrated by a clinical professor in periodontology in the Faculty of Dentistry, the University of Hong Kong. The dentist who examined periodontal status was blinded from the pain records throughout the treatment. The dentist was only in charge of the periodontal charting and was not aware of the patients’ pain experience and was not responsible for taking the cytokine samples. The baseline records were taken on the same day before bonding of the brackets. Another set of periodontal records was taken at 1 month after bonding.

Periodontal charting was carried out using a periodontal probe (CPU 15 UNC. Hu-Friedy; Chicago, IL, USA). The following parameters were recorded: plaque index (PI),^37^ GI,^27^ bleeding on probing percentage (BOP%), probing pocket depth (PPD) and gingival recession (GR). The measurements were taken at six sites for each tooth, namely mesio-buccally, mid-buccally, disto-buccally, mesio-lingually, mid-lingually and disto-lingually.

Pre-calibration was performed at baseline on approximately 10% of the sample (three patients) with a weighted kappa value of 0.75–0.80 for all of the periodontal clinical indices, indicating good intra-examiner reliability.

#### Pain assessment: visual analogue scale

The measures used for pain assessment were patients’ pain experience, pain duration and maximum pain intensity, which were evaluated using a 100-mm visual analogue scale (VAS) to record the pain within 7 days after the initial placement of the archwire. A score of 0 indicated no pain and a score of 100 indicated the most severe pain. The mark indicated by patients on the VAS was measured in mm with a ruler from the left end.

Patients who scored 0 on the VAS for the first 7 days after placement of the archwire were classified as ‘never in pain’, whereas patients who scored >0 were classified as having had a pain experience (‘pain’).

Patients were categorised into three groups based on the duration of pain after placement of the initial archwire. Group 1 comprised those in the ‘never in pain’ group, i.e. with a VAS score of 0 in the first 7 days after placement of the initial archwire. Group 2 comprised those who reported pain, i.e. VAS > 0, for 1–3 days after placement of the initial archwire. Group 3 comprised those who reported VAS > 0 for more than 3 days after placement of the initial archwire.

The maximum pain intensity of each patient was taken to be the maximum VAS score during the first 7 days following the placement of the initial archwire.

#### Biological assessment: cytokines in the GCF

GCF samples were taken from bonded incisors at baseline before bonding, 1 day after bonding and 1 week after bonding. The GCF samples were collected from the mesio-buccal and disto-buccal sites using prefabricated paper strips (Periopaper, Oraflow; New York, NY, USA), with the strips inserted into the periodontal pocket and kept in place for 30 s. Enzyme-linked immunosorbent assay (ELISA) (R&D Systems; Minneapolis, MN, USA) was performed to assess the level of PGE2 and IL-1β cytokines in the GCF fluid. Since some patients only underwent adjunctive orthodontic treatment with 2 x 4 appliances to align and intrude the displaced incisors, only incisors were chosen as the target teeth for GCF analysis.

The total cytokine concentration in each sample was assessed with a Pierce BCA Protein Assay Kit (Pierce Biotechnology; Rockford, IL, USA).

### Statistical Analysis

The dependent variables were whether the patient experienced pain, the duration of pain, the maximum pain level in the first 7 days and the cytokine concentrations. The major independent variables for this study were the PI and GI at baseline and 1 month. The categorical variables examined whether the patient experienced pain and the duration of pain. Independent-sample t-tests and one-way ANOVA were used to investigate the association of these variables with cytokine concentrations, PI and GI at baseline and 1 month. The continuous variables were the maximum pain level and the concentrations of the two cytokines. Linear regression analysis was used to investigate the association of these variables with PI and GI at baseline and 1 month. Linear regression analysis was also carried out to investigate the association between the maximum pain level and the concentrations of the two cytokines.

Statistical analysis was carried out with SPSS v 25 (IBM; Armonk, NY, USA). The level of statistical significance was set at 0.05 and the tests were two-sided.

## Results

### Characteristics of the Study Group

A total of 35 adult patients (29 women, 6 men) aged 22–59 years (mean and standard deviation [SD]: 45.62 ± 10.87 years) with treated and stabilised periodontitis participated in this study.

There was a statistically significant increase in PI from 0.41 ± 0.30 to 0.61 ± 0.32 at 1 month after bonding (p = 0.000). However, there was no statistically significant difference found in the GI (p = 0.064). In addition, there was no statistically significant difference found in any of the periodontal parameters (PPD, GR and BOP%) at 1 month after orthodontic treatment (p > 0.05) when compared to baseline (Table 1).

**Table 1:** Periodontal conditions of the subjects at baseline and 1 month after treatment

Variables	Baseline	1 month	p-value
	Mean ± SD	Mean ± SD	
**Oral hygiene**			
PI*	0.41 ± 0.30	0.61 ± 0.32	0.000*
GI	0.57 ± 0.27	0.68 ± 0.33	0.064
**Periodontal examination**			
PPD	1.58 ± 0.30	1.71 ± 0.33	0.091
GR	1.64 ± 1.39	1.53 ± 1.27	0.263
BOP %	14.20 ± 8.02	17.72 ± 9.74	0.055

*Statistically significant difference (p < 0.05) between baseline and 1 month.

Pain was experienced by 62.9% of patients in the first 7 days of treatment. A statistically significantly higher proportion of patients experienced pain on day 1 (57.1%) than day 7 (25.7%) (p = 0.004). The overall VAS score, indicating the pain level, dropped statistically significantly from 12.27 ± 17.67 on day 1 to 3.53 ± 9.72 on day 7 (p = 0.003) (Table 2).

**Table 2:** Pain characteristics of the subjects at first 7 days, day 1 and day 7 after treatment

Variables	First 7 days	Day 1	Day 7 (week 1)	p-value
Pain experience*				
Yes	22 (62.9%)	20 (57.1%)	9 (25.7%)	0.004*^a^
No	13 (37.1%)	15 (42.9%)	26 (74.3%)	
Pain level (VAS)*	–	12.27 ± 17.67	3.53 ± 9.72	0.003*

*Statistically significant difference (p < 0.05) between baseline and 1 month. ^a^Fisher’s Exact Test.

### Association Between Baseline OH and Orthodontic Pain

Patients who never experienced pain during orthodontic treatment had statistically significantly lower baseline GI than patients who experienced pain during treatment: p = 0.002; mean difference = -0.285 (95% CI -0.454 to -0.117) (Table 3). Baseline PI was not associated with the experience of pain during the first week of orthodontic treatment (p > 0.05).

**Table 3:** Association between baseline GI and whether patient experienced pain during orthodontic treatment

Variables	GI before bonding		p-value	95% CI for difference	
	Mean difference	Standard error (SE)		Lower limit	Upper limit
Pain experienced within the first 7 days after bonding					
Never pain – pain*	-0.285	0.083	0.002	-0.454	-0.117

Based on estimated marginal means. *p < 0.05 statistically significant.

The level of GI at baseline was significantly associated with the duration of pain. Patients who never experienced pain presented a lower GI at baseline than those who experienced pain for >3 days (pairwise comparison, ANOVA: p = 0.013; mean difference = 0.279 [95% CI 0.050–0.507]) and those who experienced pain for 1–3 days (pairwise comparison, ANOVA: p = 0.046; mean difference = 0.302 [95% CI 0.004–0.599]) (Table 4). The baseline PI was not statistically significantly associated with the duration of pain (pairwise comparison, ANOVA: p > 0.05).

**Table 4:** Association between baseline GI and orthodontic pain duration

Variables	GI before bonding		p-value^b^	95% CI for difference	
	Mean difference	SE		Lower limit	Upper limit
Pain duration after bonding			0.007*		
>3 days – never*	0.279	0.090	0.013*	0.050	0.507
>3 days – 1-3 days	-0.023	0.115	1.000	-0.3139	0.269
1–3 days – never*	0.302	0.118	0.046*	0.004	0.599

Based on estimated marginal means. ^b^Bonferroni adjustment for multiple comparisons: *p < 0.05 statistically significant.

Baseline GI was statistically significantly positively associated with the maximum orthodontic pain level experienced by the patients (linear regression: p = 0.022) (Table 5), while the baseline PI was not statistically significantly associated with the maximum orthodontic pain levels experienced by the patients in the first week (linear regression: p > 0.05) (Table 5).

**Table 5:** Linear regression analysis of baseline PI and GI on the maximum orthodontic pain level in the first week

Full model: R2 = 16.1%	Estimate	SE	95% CI	p-value
Baseline PI	-5.891	11.456	-29.226 – 17.445	0.611
Baseline GI*	30.495	12.670	4.687 – 56.303	0.022*

*Statistically significant at p < 0.05.

### Association Between Baseline OH and Cytokine Concentrations

Baseline GI was statistically significantly positively associated with the 1-week IL-1β (linear regression: p = 0.022) (Table 6) and PGE2 concentrations (linear regression: p = 0.009) (Table 7), i.e. higher baseline GI was associated with higher IL-1β and PGE2. However, the baseline PI was not associated with the 1-week cytokine concentrations (linear regression: p > 0.05) (Tables 6 and 7).

**Table 6:** Linear regression analysis of baseline PI and GI on 1 week IL-1ß level

Full model: R2 = 18.9%	Estimate	SE	95% CI	p-value
Baseline PI	-1.353	1.170	-3.754 – 1.048	0.258
Baseline GI*	2.726	1.118	0.432 – 5.019	0.022*

*Statistically significant at p < 0.05.

**Table 7:** Linear regression analysis of baseline PI and GI on 1 week PGE2 level

Full model: R2 = 25.4%	Estimate	SE	95% CI	p-value
Baseline PI	-4.010	2.239	-8.605 – 0.585	0.085
Baseline GI*	6.012	2.139	1.623 – 10.401	0.009*

*Statistically significant at p < 0.05.

### Association Between Cytokines and Pain Experience

Patients who reported pain in the first 7 days of orthodontic treatment had statistically significantly higher day-1 IL-1β concentrations than patients who never experienced pain (p = 0.046; mean difference = -0.435 [95% CI -0.862 to -0.009] (Table 8).

**Table 8:** Association between day 1 IL-1ß and whether patient experienced pain during treatment

Variables	Day 1 IL-1ß		p-value	95% CI for difference	
	Mean difference	SE		Lower limit	Upper limit
Pain experienced within the first 7 days after bonding					
Never pain – pain*	-0.435	0.203	0.046*	-0.862	-0.009

Based on estimated marginal means. *Statisticaly significant at p < 0.05.

The cytokine concentrations at baseline and 1 week were not associated with the patients’ pain experience, pain duration or maximum pain intensity (p > 0.05).

The first 7 days of orthodontic pain were only associated with the day-1 cytokine concentration (day-1 IL-1β), not the baseline and 1-week cytokine concentrations.

### Association Between Orthodontic Pain and 1-month Post-bonding OH

Pain experience during orthodontic treatment was not associated with the 1-month post-bonding PI and GI (p > 0.05) (Table 9), nor were duration of pain or maximum pain intensity (p > 0.05).

**Table 9:** Association between 1 month post-bonding PI, 1 month post-bonding GI and whether patient experienced pain during treatment

Variables	PI 1 month after bonding		p-value	95% CI for difference	
	Mean Difference	SE		Lower limit	Upper limit
Pain experienced within the first 7 days after bonding					
Never pain – pain	0.010	0.116	0.935	- 0.227	0.246
Variables	GI 1 month after bonding		p-value	95% CI for difference	
	Mean Difference	SE		Lower bound	Upper bound
Pain experienced within the first 7 days after bonding					
Never pain – pain	-0.179	0.113	0.124	-0.409	0.051

Based on estimated marginal means. p < 0.05.

## Discussion

This study was designed to examine two aspects of the association of OH and orthodontic pain: 1. whether the patients’ OH at the beginning of orthodontic treatment affected their level of pain during treatment, and 2. whether the orthodontic pain experienced by patients during treatment affected their post-bonding maintenance of OH. Both of these aspects are important for clinical orthodontic work, especially for patients with treated and stabilised periodontitis undergoing orthodontic treatment. However, neither has been previously well studied.

### General OH Characteristics

In this study, PI and GI were assessed to represent the patients’ OH status, and PPD, GR and BOP% were assessed to record their periodontal status. The assessment of general characteristics demonstrated that the PI increased significantly 1 month after placement of brackets and archwires when compared with baseline. This result differs somewhat from those of Cantekin et al,^4^ who found that the PI slightly decreased 1 month after bonding of brackets and increased after 1 month of orthodontic treatment. In another study, there was a statistically non-significant increase in PI 1 month after placing orthodontic brackets, but the value of PI continued to increase statistically significantly after 6 months.^7^ In our study, the PI at 1 month was statistically significantly higher than that at baseline, which was likely due to the increase in plaque-retentive areas, such as the interdental spaces caused by previous periodontal disease, in addition to the orthodontic brackets and wires. Another reason for the increase in PI could be that the patients were told not to brush their teeth 2 h before the dental appointment because plaque samples were to be collected for another study; hence, more plaque accumulated during that time due to the poor self-cleaning and higher plaque retentiveness of patients with fixed appliances. Notably, although there was a statistically significant difference in PI at baseline vs 1 month, the PI at 1 month was still below category ^1,37^ meaning that this difference was not clinically statistically significant.

The GI in this study at 1 month was not statistically significantly different from that at baseline. This result agrees with those of Dannan et al^7^ and Gujar et al,^14^ who also showed no statistically significant change in GI up to 1 month after orthodontic treatment when investigating the OH of healthy patients without periodontitis. The fact that there was no statistically significant difference in GI between baseline and 1 month indicates that the levels of OH were well maintained in this group of treated and stabilised periodontitis patients undergoing orthodontic treatment. In addition, there was no statistically significant change in any of the periodontal parameters in this study when comparing the charting at the baseline to the charting at 1 month. This is in agreement with the findings of a doctoral thesis, showing that orthodontic treatment did not improve or exacerbate the status of periodontally compromised dentitions.^42^ Overall, with the minor increase in the PI and the statistically non-significant increase of the GI and periodontal parameters, it can be concluded that the patients’ OH levels in this group were well maintained throughout the assessment period during orthodontic treatment.

### General Pain Characteristics

The overall percentage of patients who experienced orthodontic pain in the first 7 days of treatment was 62.9%, which is similar to a previous finding that 58.5% of patients experienced pain during orthodontic treatment.^24^ However, the proportion of patients reporting pain at day 7 in this study was much lower than that reported in other studies, which found that 40% of patients reported orthodontic pain after 1 week.^9,28^ Adolescents have been shown to generally experience higher levels of pain than adults,^23^ suggesting that the lower proportion of patients experiencing pain at day 7 in this study than in some previous studies could be due to the recruitment of adult subjects here, as opposed to the adolescents studied in previous research. In this study, there was a statistically significantly higher proportion of patients experiencing pain on day 1 (57.1%) than day 7 (25.7%). Another finding was that the pain level dropped statistically significantly from day 1 to day 7 after the commencement of orthodontic treatment. This concurs with previous studies showing that orthodontic pain usually peaks after 1 day and gradually diminishes from 3 to 7 days.^9,28^

### Baseline GI and Orthodontic Pain

It was found that baseline GI was positively associated with orthodontic pain experience, pain duration and maximum pain intensity. First, patients who experienced no pain in the first 7 days of orthodontic treatment had significantly lower GI than those who did. Second, patients who experienced pain from day 1 to day 3 and who experienced pain for more than 3 days also had significantly higher GI than those who never had pain. Third, higher baseline GI was associated with experiencing a higher maximum orthodontic pain intensity during treatment. Although no other study has reported the effects of OH on orthodontic pain, one study examined the relationship between gingival inflammation and the pain caused by other mechanical stimuli, e.g. periodontal probing and scaling. In that study, it was found that patients with greater gingival inflammation usually experienced more pain and discomfort from periodontal probing and scaling.^16^ It has been demonstrated that inflammation modifies the responses of mechanical nociceptors in the gingiva.^6,10^ A higher GI indicates more gingival inflammation, and thus more inflammatory mediators are released, which sensitise the nociceptors in the inflamed gingiva. When orthodontic force is applied in patients with sensitised nerve endings, they will experience a higher intensity of pain. This may be why the patients in our study with higher GI had a longer duration and higher intensity of pain.

To further explore these associations at the biological level, we collected GCF samples from the patients to assess the concentration of PGE2 and IL-1β cytokines at baseline, day 1 and day 7, and their relationship with baseline OH conditions and the orthodontic pain experience. Although periodontal diseases are initiated by plaque bacteria, the host response is believed to play a major role in the breakdown of connective tissue. Microbial antigens in the plaque elicit inflammatory and immune reactions. The host response differs among individuals, depending on the concentrations of cytokines and the inflammatory cell response. IL-1β and PGE2 play a pivotal role in mediating soft tissue and bone resorption.^22,38^ Mechanical force can also cause an increase in the concentrations of cytokines such as PGE2 and IL-1β in the GCF of teeth undergoing orthodontic movement.^41,43^ Orthodontic forces applied to the teeth lead to the development of areas of compression and tension in the periodontal ligament, which alter the blood flow and the release of cytokines during movement of the tooth. At the same time, the cytokines may also elicit a hyperalgesic response.^23^ Several inflammatory cytokines that are released during periodontal inflammation, such as IL-1β and PGE2, are also released during orthodontic tooth movement. A study^28^ investigating the mechanism of orthodontic pain found that the cytokines involved in bone remodelling also function as mediators of the hyperalgesic response; IL-1β and PGE2 directly and indirectly amplified the local inflammation and stimulated the sensory nerve endings to generate painful sensations.

### Association Between GCF and Pain

This study showed that the concentration of IL-1β on day 1 was associated with the pain experienced during orthodontic treatment, as patients who experienced pain had statistically significantly higher concentrations of IL-1β at day 1 than those who did not. However, the baseline and 1-week cytokine concentrations (IL-1β and PGE2) were not associated with whether the patient experienced pain during treatment, the duration of pain or the maximum pain intensity. A possible reason for the significant result being found solely on day 1 is that the cytokine concentrations usually peak 24 h after the initial orthodontic force is applied, then return to the baseline values after approximately 7 days.^13,25,43^ Thus, the pain experienced during orthodontic treatment is mainly reflected by the cytokines at day 1 and not at baseline or day 7.

### Baseline GI and GCF

Baseline GI in this study was not associated with baseline cytokine concentration. However, baseline GI was statistically significantly correlated with the concentration of IL-1β and PGE2 at 1 week. In pathophysiological states, chronic inflammation enhances the expression of pro-inflammatory cytokines.^36^ The increase in IL-1β and PGE2 concentrations at 1 week for patients with higher baseline GI might have been caused by the enhanced and sustained immune reaction resulting from the mechanical stimulation of pre-existing inflamed gingival tissue. Therefore, the positive correlation between baseline GI and 1-week cytokine concentrations could be due to the dominating contributions of the patient’s pre-existing periodontal component, rather than mechanical stimulation by orthodontic force.

### Baseline OH and Pain

This study showed that unlike baseline GI, baseline PI was not associated with the experience, intensity or duration of orthodontic pain. We attribute this to the fact that the PI can only reflect the OH condition at the time of examination, whereas the GI reflects the OH level of the patient at least a few days before examination. In other words, the PI cannot distinguish patients who practise meticulous OH every day throughout orthodontic treatment from those who tend to only clean the teeth well immediately before the orthodontic appointment. It is well accepted that the most common cause of inflamed gums is plaque. If plaque is not removed, the gums will become inflamed within a few days.^17^ In addition, all of the patients were asked not to brush their teeth 2 h before the dental appointment, as mentioned above. Hence, the PI may not truly reflect the patients’ general OH level.

### Orthodontic Pain and OH Maintenance

To understand the association between OH and orthodontic pain, besides the effects of baseline OH on orthodontic pain as discussed above, another important aspect is whether orthodontic pain affects the standard of OH practice during orthodontic treatment. Good OH maintenance is a key element of successful orthodontic treatment and is particularly important in patients with previous periodontitis. Poor plaque control with gingival inflammation leads to periodontal attachment loss and bone loss, and in turn to an overall reduction of periodontal support.^8^ As fixed orthodontic treatments are reported to be plaque-retentive, and patients undergoing orthodontic treatment are at higher risk of gingivitis, the key to prevent this problem is adequate plaque control.^3,26,40^

No previous study has directly examined the association between orthodontic pain and post-bonding OH level. Goldstein and Gilbert^11^ stated that chronic pain has an emotional, psychological and physical impact on the way a person conducts his/her daily life. This includes poor personal and dental hygiene.^11^ Cavalehiro et al^5^ also showed that dental pain affects talking, cleaning teeth/gums and eating, but they did not investigate orthodontic pain. Sergl et al^35^ found that orthodontic pain led to poor overall compliance, where poorer OH was one of the factors included when assessing the overall compliance, but the authors did not specifically measure the association between orthodontic pain and the level of OH of patients. Hence, the present study provided new evidence regarding this previously unanswered question by comparing orthodontic pain with the level of post-bonding OH practice. It was established that no association existed between orthodontic pain and the post-bonding OH practice of the patients, as neither the experience and duration of pain nor the maximum pain intensity was associated with the 1-month post-bonding PI and GI. This study also reviewed the patients’ post-bonding OH at 1 month rather than 1 or 2 weeks after bonding, to allow sufficient time for the patients to become accustomed to performing dental cleaning with the fixed orthodontic appliances and to assess their genuine periodontal condition more accurately. The results indicated that the discomfort/pain caused by orthodontic force was not an inhibitory factor for patients’ OH maintenance. A possible explanation is that the intensity of orthodontic pain is relatively low and that the discomfort is short-lived.

### Limitations

It must be noted that all of the patients in this study had treated and stabilised periodontitis and had been under supportive periodontal care. These patients thus had good awareness of, and high motivation to achieve, satisfactory OH maintenance. Therefore, their PI and GI were well controlled before and during orthodontic treatment. Hence, the results of this study may not be able to predict the genuine pain level of patients whose OH is poorly maintained during orthodontic treatment. Moreover, the patients studied here may have been more conscious of good OH, and motivated to maintain this, than regular orthodontic patients. Therefore, the results may not be applicable to all orthodontic patients. Further study is needed to identify the association between OH and orthodontic pain in a more general range of subjects undergoing orthodontic treatment.

This study also took into account some possible confounding factors, including age, gender and dental crowding and performed the statistical analysis with the adjusted models. However, no statistical significance was found using the adjusted models, which could be due to the limited sample size. This agrees with the results of other studies showing that there was no statistically significant association between pain perception and age, gender and the extent of tooth displacement.^9,20,31^ Nevertheless, a larger sample size is needed for future studies.

Although the sample size of 35 is relatively small, it met the minimum number of 30 subjects calculated from the sample size calculation using G*Power version 3.1.9.2 (Franz Faul). There are several studies that have dealt with orthodontic treatment in subjects susceptible to periodontitis using inclusion criteria similar to those in this study. Their sample sizes range from 28-30 subjects, fewer than the number of subjects recruited in this study.^29,30,33^ Future studies with larger sample sizes are required.

This study provides the first clinical evidence of the association between OH and orthodontic pain from two aspects, i.e. the association of OH status before treatment and the orthodontic pain level, and that of the orthodontic pain and the level of post-bonding OH maintenance among a group of patients with treated and stabilised periodontitis. A positive association was found between the baseline GI and orthodontic pain. However, no association was found between orthodontic pain and the post-bonding OH practice. The results indicate that the better patients maintain their OH, the less orthodontic pain they are likely to experience during orthodontic treatment, and that the discomfort during orthodontic treatment is not a cause of poor OH maintenance after the bonding of orthodontic appliances.

## Conclusion

The increased level of gingival inflammation accounts for the longer duration and higher intensity of orthodontic pain shortly after force loading in treated and stabilised periodontal patients. It indicates that oral hygiene instructions and supportive periodontal care are of great importance, prior to and during adjunctive orthodontic treatment in periodontally compromised individuals.
